# Deltamethrin Residues in Milk and Cheese of Lactating Goats (*Capra hircus*)

**DOI:** 10.3390/molecules24030517

**Published:** 2019-01-31

**Authors:** Raffaele Marrone, Abilasha Ramkumar, Giorgio Smaldone, Domenico Rufrano, Claudia Chirollo, Vincenzo Veneziano, Martin Danaher, Aniello Anastasio

**Affiliations:** 1 Department of Veterinary Medicine and Animal Production, University of Naples ‘‘Federico II’’ Via F. Delpino, 1-80137 Naples, Italy; giorgio.smaldone@unina.it (G.S.); c.chirollo@gmail.com (C.C.); vinvene@unina.it (V.V.); anastasi@unina.it (A.A.); 2 Food Safety Department, Teagasc Food Research Centre Ashtown, Dublin 15, D15 DY05 Dublin 15, Ireland; abilasha.ramkumar@teagasc.ie (A.R.); Martin.Danaher@teagasc.ie (M.D.); 3 Research Unit for the Extensive Animal Husbandry, CRA ZOE, Muro Lucano (PZ), 85054 Muro Lucano, Italy; drufrano@tiscali.it

**Keywords:** ectoparasites, pyrethroid, withdrawal period, UHPLC-MS/MS

## Abstract

The distribution of pyrethroid insecticide deltamethrin (DLM) in goat milk and cheese (caciotta) following pour-on administration at the sheep dosage (DLMS-10 mL/60 kg body weight) and double dosage (DLMD-20 mL/60 kg body weight) was studied. DLM concentrations were measured in milk collected from study animals (No.14) before treatment and at 2, 4, 8, 12, 16, 24, 30, 36, 48, 56, until 168 h (7 days) post treatment and in caciotta cheese at 12 and 24 h post treatment. At both dosages, the maximum level of DLM residues in goat milk and cheese was below the maximum residue limit (MRL) of 20 μg kg^−1^ established for bovine milk (EU No 37/2010) at all time points. However, in terms of public health, higher DLM residues in cheese show that further specific studies should be performed on double dosage efficacy and pharmacokinetic and pharmacodynamics properties of ectoparasites in lactating goats.

## 1. Introduction

Goat milk and its products play a significant role in human nutrition [1]; it is beneficial to health because it is easily digested by lactose intolerant people [2]. Moreover, goat dairy products have a typical flavor and aroma and are probably the last alternative to standardized milk products [3]. Ectoparasites often cause skin lesions in ruminants and heavy infestation has a negative impact on the general animal health status and dairy efficiency [4]. Arthropod ectoparasites that have a major impact on the productivity and welfare of goats are lice (Damalina caprae, Linognathus stenopsis) and ticks (Rhipicephalus spp., Haemaphysalis spp.) [5]. Different classes of drugs to control parasites used in cattle and sheep are commonly extrapolated to goats without optimization of dosage regimens and determination of pharmacokinetic and pharmacodynamic properties [6]. Therefore, irrespective of the dosage used, more accurate withdrawal periods defined by scientific experimentation are required to ensure that dairy products from treated animals are safe for human consumption.

The most commonly used insecticides in ruminants are mainly topically used as pour-on formulations [7]; among these, an important role is played by pyrethroids, which are synthetic analogs of pyrethrins [8]. Synthetic pyrethroids are more resistant to degradation and are highly active against a broad spectrum of ectoparasites [9]. Deltamethrin (DLM), [(S)-α-cyano-d-phenoxybenzyl-(1R,3R)-e-(2,2dibromovinyl)-2,2dimethylcyclopropane-1-carboxylate], is a type II pyrethroid insecticide used to control ectoparasites in sheep and cattle. Its applications include pour-on formulations, ear tags, dips and sprays. DLM is a relatively potent neurotoxin, the toxicity of DLM has been extensively examined and a recent study shows that DLM increases toxicity of rat blood through the oxidative stress of erythrocytes [10]. In Italy, DLM has been marketed as a pour-on formulation to prevention and treatment of flies, lice, keds and ticks on cattle and sheep with different dose milk-withdrawal time. However, detailed pharmacokinetic studies were performed for DLM in sheep and cattle, in literature there are few data on goats. For example, Juliet et al., 2001 showed that in black Bengal goats maximum blood concentration of deltamethrin was recorded at 0.5 min [11]. The aim of the present study was to investigate DLM residues in goat milk and evaluate its levels in cheese of lactating goats treated pour-on with a single or a double sheep dosage of ectoparasites drug. An analytical method that uses Quick, Easy, Cheap, Effective, Rugged, and Safe (QuEChERS) based sample preparation and detection by UHPLC-MS/MS has been developed and validated in-house to support this study [12].

## 2. Results and Discussion

The analytical method for the detection of DLM was fully validated prior in milk and cheese analysis. 

### 2.1. Analytical Method Validation

The limit of detection and limit of quantification (LOD and LOQ) are defined as the lowest concentration of the analyte with S/N = 3 and S/N = 10, respectively. S/N > 10 was obtained for all the measurements. Transitions and cone voltages for DLM and IS are reported in Table 1. According to Martinez et al. 2006 [13], when DLM was subjected to positive ion electrospray ionization with ammonium acetate, the ammoniated molecule ions were produced [M + NH_4_]^+^ and the most abundant transitions were at *m/z* 522.7 –––> 280.8 (quantification ion) and *m/z* 524.7 –––> 282.8 (confirmatory ion). The method was applied to fortified negative goat milk samples from different animals and the trueness, sensitivity and specificity were calculated.

**Figure 1:**
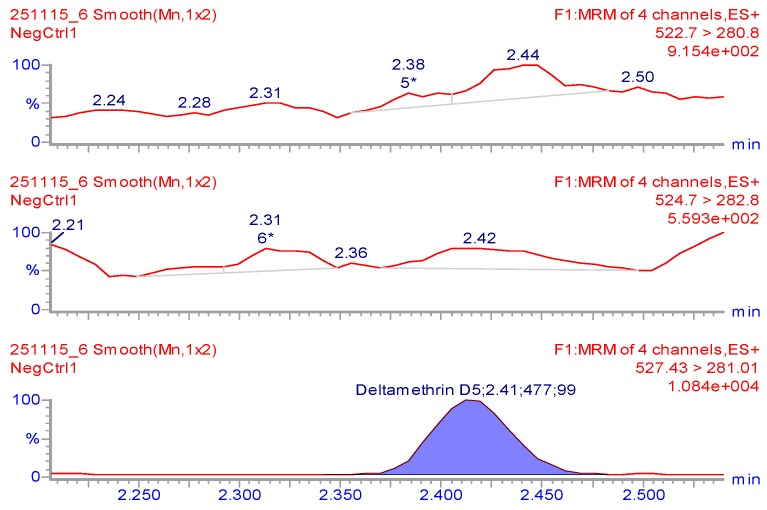
MRM of negative goat milk samples spiked with internal standard (deltamethrin-D5).

**Figure 2:**
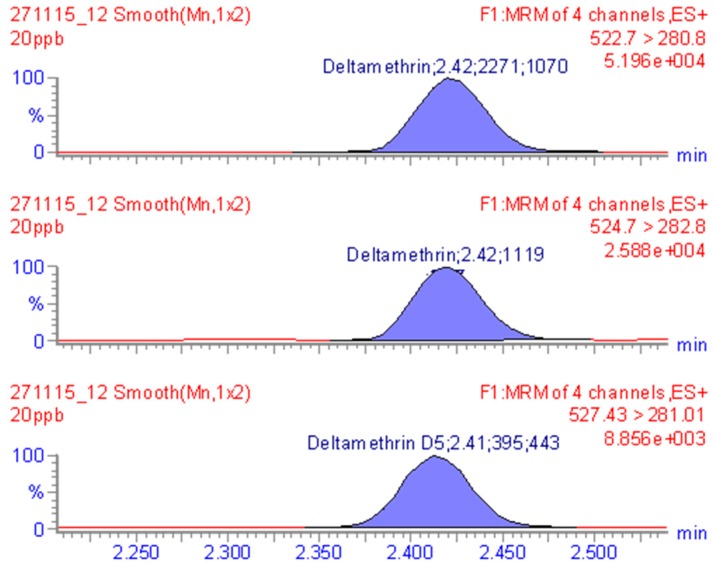
MRM chromatogram of goat milk sample fortified with 20µg kg^−1^ of deltamethrin standard.

Chromatographic traces of DLM at specific retention time are reported in Figure 1. Since a maximum residue level (MRL) has not been set for DLM in goat milk, this value was set at the middle of calibration curve designed to the current MRL for bovine milk (20 μg kg^−1^) [14] (Figure 2). 

In order to determine the accuracy and linearity of the method, aliquots of the drug-free control samples of goat milk (organic goat milk) and cheese were spiked with DLM at seven levels: 1, 2, 4, 10, 20, 30 and 40 μg kg^−1^.

Mean calibration curves in milk and cheese were linear (R^2^ > 0.998). The limit of quantitation of the method, as determined from the lowest standard on the calibration curve was 1 μg kg^−1^. Intra-assay variation was determined by analyzing five samples within a single run; reproducibility (RSDs) were typically less than or equal to 6%. Mean trueness of the analytes was between 95 and 100%. Mean recovery of analytes was 95% or higher (Table 2). Inter-assay variation was determined by analyzing samples on five different occasions, to evaluate the run-to-run variation in the method (Table 3). CCα and CCβ were calculated according to calibration curve procedure [15] and the values were 21.2 and 23.3 μg kg^−1^, respectively. MRM chromatograms of goat milk samples fortified with DLM at 1, 2 and 4 μg kg^−1^ had enough scans across the peak (12–15) and acceptable signal-to-noise ratio for applied transitions.

### 2.2. Dermal Safety and Persistence of DLM in Goat Milk and Cheese

During the study, the DLM pour-on formulation (at the dose rate of sheep and double dosage: 75 and 150 mg/animal respectively) was well tolerated by all the treated animals with no adverse reactions. The dermal safety study on the DLM-groups showed the absence of adverse effects caused by the treatment (Draize score = 0) in all the goats. Table 4 and Table 5 show the results of the DLM residues (μg kg^−1^) in milk and cheese samples, respectively.

DLM residues were not detected in milk until 8 h post treatment and levels in milk ranged from 0.16 to 1.67 μg kg^−1^ after DLMS dose and from 0.17 to 1.74 μg kg^−1^ after DLMD dose. The highest levels were determined in samples collected at 30 h post treatment at DLMS dose; when DLMD dose was used, highest levels were detected at 36 h post treatment. The concentrations of DLM decreased progressively until 120 h after treatment, where they were less than the detection limit at both dose rates. There were no differences (*P* < 0.05) in the average of DLM milk residue for time sampling among the two dosage (DLMS and DLMD).

DLM residues measured in cheese, obtained with pooled milk, were higher than those recovered in milk and ranged from 5.75 to 10.3 μg kg^−1^ for DLMS dose and from 9.45 to 11.15 μg kg^−1^ for DLMD dose; highest levels were observed after 24 h for both treatments. In cheese, differently from milk, DLM residues following DLMD dose, at 12 h, were significantly higher (5.75 µg kg^−1^ vs. 9.45 µg kg^−1^) than DLMS dose whilst at 24 h values were similar at both treatments. Significant differences (*P* < 0.05) between different dosage and sampling time were found.

There are few studies on the fate of DLM in lactating species. According to Venant et al. (1990) [16], after a ‘pour-on’ application of DLM (0.1 g and 1 g) in lactating dairy cows, drug residues in milk were less than 1% of the treatment dose, and maximum levels were reached after 2 days (9 μg L^−1^ for 0.1 g DLM and 53 μg L^−1^ for l g DLM). Results of present studies showed that, considering a similar administration dose (0.15 g DLMD vs. 0.1 g of Venant et al. study), the time to reach the maximum level was slightly longer (36 h vs. 33 h) compared to the trials conducted in the cattle and concentrations were significantly different (1.3 μg L^β1^ vs. 9 μg L^−1^). 

Other drugs belonging to the same pyrethroid insecticides group of DLM, such as α-cypermethrin, showed a different fate. Smaldone et al. (2013) [17] found α-cypermethrin in buffalo milk levels were above the maximum residual limit of 20 μg kg^−1^ established for bovine milk [14] after pour-on treatment following manufacturers recommended dose, while Chirollo et al. (2014) [18] reported a rapid depletion of α-cypermethrin concentrations in milk of lactating donkeys. Moreover, it is important to note that other antiparasitic substances have a different behavior in goat compared to cow and sheep. Anastasio et al. (2005) [19] showed that eprinomectin concentrations detected in goat milk following pour-on administration were different from those observed in dairy cattle. Data on doramectin, other macrocyclic lactones administered pour-on in goat, showed similar metabolic and disposition profile compared to deltamethrin since goats show rapid depletion and short drug half-lives compared to sheep and cattle [20].

The Committee for Medicinal Products for Veterinary Use defines goats as a minor species [21]; in this species antiparasitic drugs are often administered with extra-label dosages and the withdrawal intervals usually are based on recommendations for cattle and sheep because very few drugs have specific indications on the label about goats. According to Jacquet and Dorchies, 2002 [22] goats have a greater capacity to metabolize many drugs than other ruminants; therefore, it is essential to administer higher doses for shorter intervals to ensure an effective control of ectoparasites [23]. Although studies have determined the depletion and behavior of DLM in ruminants, there is scarce information on specific behavior of lactating goats.

There are no studies on the fate of pyrethroids in dairy products. The finding of this research could be useful to characterize the lipid solubility property of DLM that may concentrate in curd of milk-producing animals during manufacturing process.

## 3. Materials and Methods

### 3.1. Chemicals and Apparatus

DLM analytical standard (Pestanal Sigma-Aldrich, Seelze, Germany, 99.7%) was purchased from Sigma-Aldrich, (Dublin, Ireland) and deltamethrin-D5 internal standard (98%) was obtained from Toronto Research Chemicals (Toronto, Canada). SpS-grade acetonitrile and methanol were obtained from Romil Chemicals (Dublin, Ireland). Sigma-Aldrich supplied anhydrous magnesium sulphate (MgSO_4_) and ammonium acetate (C_2_H_7_NO_2_) (purum p.a. grade). Preweighed mixture of 1.5 g anhydrous MgSO_4_ and 0.5 g of C_18_ bulk sorbent in 50 mL centrifuge tubes were obtained from UCT, Inc. (Bristol, PA; USA). Sodium chloride (>99.5%) was obtained from AppliChem GmbH (Darmstadt, Germany). Ultra-pure water (18.2 MΩ) was generated in-house using a Milli-Q Plus water purification system. PTFE syringe filters (13 mm, 0.2 μm) were obtained from Agilent Technologies (Cork, Ireland). An ultra-turrax probe blender from IKA (Staufen, Germany), a Mistral 3000i centrifuge from MSE (London, UK), a VWR International (Dublin, Ireland) during sample preparation multi-tube vortexer, and a Transonic 780 LH ultrasonic bath from Mason Technology (Dublin, Ireland). For a chromatographic separation was used an “Acquity UHPLC system” (Waters, Milford, MA, USA). The analyte was separated using the chromatographic conditions developed previously [15]. Deltamethrin residues were detected using a Quattro Premier triple quadrupole mass spectrometer (MS) equipped with an electrospray ionization (ESI) probe (Waters, Milford, MA, USA). To minimize contamination of the MS source, the eluent flow was diverted to waste for 2 min after sample injection followed by a second divert (after 2.5 min) to waste prior to the next chromatographic sequence. Capillary voltage was set at 3.0 kV. Analyte-specific instrument parameters (i.e., collision energy, capillary voltage and cone voltage) were optimized prior to analysis through a teed infusion of 1 μg mL^−1^ deltamethrin standard solution. The inter-channel delay and inter-scan delay were set to obtain maximum instrumental response.

### 3.2. Extraction, Clean-Up Procedure and Validation

A QuEChERS based approach was adapted to isolate deltamethrin residues from goat milk and cheese samples. Milk (10 ± 0.1 g) and cheese samples (1 ± 0.01 g) were weighed into 50 mL polypropylene centrifuge tubes (Sarstedt, Wexford, Ireland). The samples were then fortified with internal standard (IS) solution (200 μL) and cheese samples were diluted with 9 mL of water followed by homogenisation using an ultra-turrax probe blender. The samples were extracted with acetonitrile MeCN (10 mL) by shaking vigorously for 1 min in the presence of MgSO_4_ (4 g) and NaCl (1 g) to induce phase separation. Samples were subsequently centrifuged (2842× *g*, 12 min) and the supernatant was transferred to dispersive solid phase extraction (d-SPE) tubes containing pre weighed C_18_ bulk sorbent and MgSO_4_. The samples were then vortexed for 1 min and centrifuged (2842× *g*, 10 min). The obtained extract was filtered through 0.2 μm PTFE syringe filters and 5 µL was injected onto the UHPLC-MS/MS system.

Stock standard solution of DLM and DLM-D5 were prepared at concentrations of 2000 μg mL^−1^ and 100 μg mL^−1^, respectively, in ethanol. DLM-D5 was used as internal standard (IS). Intermediate standards were prepared in acetonitrile at 100 μg mL^−1^ and 1 μg mL^−1^ for DLM and IS, respectively. Working standard fortification solutions at concentrations of 0.05 (std 1), 0.1 (std 2), 0.2 (std 3), 0.5 (std 4), 1 (std 5), 1.5 (std 6) and 2 (std 7) μg mL^−1^ were prepared in acetonitrile. 

Commercial goat milk purchased in a supermarket, found not to contain any detectable DLM residues, was used as the negative control. Matrix calibration curves were prepared by fortifying negative milk samples prior to extraction with 200 μL of each calibration standard solution (std 1–7) to give matrix concentrations of 1, 2, 4, 10, 20, 30 and 40 μg kg^−1^ in the matrix. Recovery was assessed by analyzing milk sample extracts spiked post-extraction with DLM at concentrations of 2 and 30 μg kg^−1^ in duplicate. 

The following performance parameters were investigated: intra-assay variations, inter-assay variations, linearity, recovery, decision limit (CCα) and detection capability (CCβ). The accuracy and precision of the method were determined using negative goat milk samples fortified at 0.5, 1 and 1.5 times the MRL level of 20-μg kg^−1^, which was listed by Commission Regulation (EU) No. 37/2010 for DLM residue in bovine milk [14].

### 3.3. Study Farm, Experimental Animals and Sampling

The study was conducted in an experimental small ruminant farm (Extensive Animal Husbandry, CRA ZOE) located in southern Italy, Potenza province (40°21′ N and 15°30′ 25″ E). For this study were selected fourteen Saanen lactating goats (Capra hircus), with a mean age of 3 ± 2 years and a mean bodyweight of 50 ± 10 kg. The animals were fed with concentrated food, with hay during the study period, and were milked twice a day; water was given ad libitum throughout the course of the study. Research animals were assigned at random to the following treatment groups of 7 animals each: DLM sheep dose group (DLMS-group), DLM sheep double dose group (DLMD-group). Throughout the experimental period each animal was isolated from the others in a single indoor box (6.25 m^2^) to avoid physical contact and licking. On day 0, the research animals received DLM pour-on (Butox^®^ 7.5 mg mL^−1^, MSD Animal Health, Italy) at the manufacturers recommended sheep dose of 10 mL per animal for the the DLMS group, and at the double sheep dose of 20 mL per animal for the DLMD group. The pour-on formulation was applied topically with a syringe along the midline of the back from the withers to the trailhead. Milk samples were collected from each of the study animals (*n* = 14) before treatment (day 0) and at 2, 4, 8, 12, 16, 24, 30, 36, 48, 56, 96, 120, 144 and 168 h post treatment. The total daily production of the treated DMLS and DMLD goats was collected separately at the day 0, 12 and 24 h after treatment and kept at 4 °C. The milk was processed in the same time points to produce caciotta cheese with ripening period of 30 days. The processing techniques of caciotta cheese are described in Rubino et al., 2004 [24]. Raw milk was heated to 36 to 38 °C and a paste of kid rennet was added. On coagulation, the curd was broken with a wooden stick to granules the size of nuts. Next, the curd was gathered on the bottom of the vat, put into small wicker baskets, and set to dry on a tilted wooden table. Salt was rubbed on the cheese once a day for 3 days. After 7 days, the forms were dipped into fresh water for 24 h, dried, and aged 30 days on wooden tables. For individual milk and cheese samples at sampling time, 2 samples were analyzed in duplicate; results are means and standard deviations. The study animals were observed by a veterinarian coauthor periodically for the first day of the study (three times, every 4 h) for possible adverse reactions, then daily for 1 week until the end of the trial. Skin examination was carried out using a modified Draize scoring system [25]. In short, a score of 0–4 was applied to each treated animal in respect of: (a) erythema and eschar formation, (b) edema formation, and (c) hair loss (at site of application), with the score 0 indicating the absence of change and the scores 1–4 indicating increasing severity of dermal change. The investigation was approved by the Animal Ethics Committee of the University of Naples, Federico II (Naples, Italy).

### 3.4. Statistical Analysis

ANOVA was used to compare concentrations of DLM between different dosage (DLMS and DLMD groups) in milk and cheese samples and different steps of time sampling. Statistical significance was considered to be at the 0.05 level. All of the statistical analysis was run using SPSS, Version 11.5, SPSS Inc., Chicago, IL, USA. 

## 4. Conclusions

The findings of the present study show that the use of DLMD, with higher antiparasitic activity, pose no residues problems for goat milk and dairy products. In fact, highest levels of DLM in milk and cheeses after administration of 20 mL/60 kg body weight of DLM were always below the MRL of 20 μg kg^−1^, which has been set for bovine milk by EU Commission Regulation 37/2010. Other specific studies must be conducted to provide more information on double dosage efficacy and pharmacokinetic and pharmacodynamics properties of drugs against ectoparasites in goats since they are milk-producing animals. This could have important implications in terms of public health and trade of goat milk and byproducts.

## Figures and Tables

**Table 1:** Multiple reaction monitoring (MRM) conditions for deltamethrin and deltamethrin-D5 (IS).

Compound	Parent Ion (*m/z*)	Daughter Ion (*m/z*)	Dwell Time (s)	Cone Voltage (V)	Collision Energy (eV)	Retention Time (min)
Deltamethrin	522.7	280.8	0.1	20	16	2.4
	524.7	282.8	0.1	20	16	2.4
Deltamethrin-D5 (IS)	527.4	281.1	0.1	20	16	2.4

**Table 2:** Intra-assay variations in goat milk samples (*n* = 5, fortified at 10, 20 and 30 µg kg^−1^).

Deltamethrin (µg kg^−1^)			
	10	20	30
	9.4	20.4	29.5
	10.5	20.1	31.4
	10.0	20.3	34.4
	9.9	20.3	31.2
	10.9	19.5	30.3
Mean	10.2	20.1	31.4
CV	5.6	1.8	6.0
Recovery %	102	101	105

**Table 3:** Inter-assay variations (%) in goat milk samples (*n* = 2, spiking at 10, 20 and 30 µg kg^−1^).

Day	Deltamethrin (Mean (*n* = 2))		
	10 µg kg^−1^	20 µg kg^−1^	30 µg kg^−1^
1	10.0	19.9	30.9
2	9.5	20.5	29.8
3	9.8	20.9	34.2
4	9.3	19.2	27.5
5	9.6	18.2	30.7

**Table 4:** Mean ± SD of DLM residue (μg kg^−1^) in goat milk following pour-on DLM sheep dose (DLMS-group) and DLM sheep double dose (DLMD-group) treatment (groups of 7 animals each).

	**Sampling Time (h)**													
**DLMS-Group**	**2**	**4**	**8**	**12**	**16**	**24**	**30**	**36**	**48**	**56**	**96**	**120**	**144**	**168**
DLM (μg kg^−1^)														
Mean	-	-	<1	<1	1.15	1.30	1.67	1.32	<1	1.11	<1	<1	-	-
SD	-	-	0.42	0.70	0.97	0.94	1.25	1.25	0.55	0.53	0.35	0.33	-	-
	**Sampling Time (h)**													
**DLMD-Group**	**2**	**4**	**8**	**12**	**16**	**24**	**30**	**36**	**48**	**56**	**96**	**120**	**144**	**168**
DLM (μg kg^−1^)														
Mean	-	-	<1	<1	1.11	1.71	1.63	1.74	1.06	1.62	<1	<1	-	-
SD	-	-	0.29	0.86	1.34	1.41	1.52	1.20	1.06	1.33	0.44	0.33	-	-

**Table 5:** Mean ± SD of DLM residue (μg kg^−1^) in goat cheese elaborated with pooled milk (groups of 7 animals each) following pour-on DLM sheep dose (DLMS-group) and DLM sheep double dose (DLMD-group) treatment.

DLM (μg kg^−1^)	Sampling Time (h)		
Group	0	12	24
DLMS	-	5.75	10.30
SD	-	±0.91	±0.70
DLMD	-	9.45	11.15
SD	-	±1.20	±1.34
